# Norcholic Acid Promotes Tumor Progression and Immune Escape by Regulating Farnesoid X Receptor in Hepatocellular Carcinoma

**DOI:** 10.3389/fonc.2021.711448

**Published:** 2021-11-23

**Authors:** Yihang Gong, Kun Li, Yunfei Qin, Kaining Zeng, Jianrong Liu, Shaozhuo Huang, Yewu Chen, Haoyuan Yu, Wei Liu, Linsen Ye, Yang Yang

**Affiliations:** ^1^ Department of Hepatic Surgery and Liver Transplantation Center, The Third Affiliated Hospital of Sun Yat-sen University, Guangzhou, China; ^2^ Guangdong Provincial Key Laboratory of Liver Disease Research, The Third Affiliated Hospital of Sun Yat-sen University, Guangzhou, China; ^3^ Department of Biotherapy Center, The Third Affiliated Hospital of Sun Yat-sen University, Guangzhou, China; ^4^ Department of General Surgery, The Third Affiliated Hospital, Sun Yat-sen University, Guangzhou, China

**Keywords:** hepatocellular carcinoma (HCC), bile acids, exosomes, immune microenvironment, Farnesoid X receptor

## Abstract

Accumulating evidence shows a close association between various types of bile acids (BAs) and hepatocellular carcinoma (HCC), and they have been revealed to affect tumor immune response and progression mainly by regulating Farnesoid X receptor (FXR). Nevertheless, the roles of Norcholic acid(NorCA) in HCC progression remain unknown yet. In this study, herein we demonstrate that NorCA can promote HCC cell proliferation, migration and invasion through negatively regulating FXR. Additionally, NorCA can increase PD-L1 level on the surfaces of HCC cells and their exosomes, and NorCA-induced exosomes dramatically dampen the function of CD4^+^T cells, thereby inducing an immunosuppressive microenvironment. Meanwhile, a negative correlation between PD-L1 and FXR expression in human HCC specimens was identified, and HCC patients with FXR^low^PD-L1^high^ expression exhibit a rather dismal survival outcome. Importantly, FXR agonist (GW4064) can synergize with anti-PD-1 antibody (Ab) to inhibit HCC growth in tumor-bearing models. Taken together, NorCA can promote HCC progression and immune invasion by inhibiting FXR signaling, implying a superiority of the combination of FXR agonist and anti‐PD‐1 Ab to the monotherapy of immune checkpoint inhibitor in combating HCC. However, more well-designed animal experiments and clinical trials are warranted to further confirm our findings in future due to the limitations in our study.

## Introduction

Bile acids (BAs) are metabolites generated in the liver and synthesized from cholesterol *via* both the nonclassical and classical pathways, which are under the control of specific enzymes (1). The dysmetabolism of BAs can promote the development of HCC associated with obesity or fatty liver (2). In a mouse model of nonalcoholic steatohepatitis-associated HCC, the accumulation of secondary BAs led to hepatocyte inflammation and contributed to carcinogenesis (3). When the BA pool was reduced by administering 2% cholestyramine in food, the sizes of malignant lesions were significantly decreased (4). The farnesoid X receptor (FXR) modulates BAs homeostasis *via* enterohepatic circulation (5). In the liver, FXR activates small heterodimer partner (SHP) expression, thereby suppressing the level of the cytochrome P450 A1 enzyme, which catalyzes the *de novo* synthesis of BAs from cholesterol (6). The FXR-KO model causes dysregulation of BAs metabolism and spontaneous hepatocarcinogenesis (7). Depletion of FXR is the causative factor for the induction of chronic inflammation, hepatocyte damage and the development of HCC (8, 9). Furthermore, FXR is considered to be a modulator of immune responses in a subset of immune disorders. Increased FXR modulates CD8^+^ T cell metabolism (10) and downregulates the expression of inflammatory regulators (IFNγ, IL6, and IL1β) in a colitis mouse model (11).

Studies on BAs in HCC have focused on the direct effects of BAs on tumor cells, while the role of BAs in the cross talk between HCC and immune cells remains unclear. Recent studies have reported that Exos play pivotal roles in the cell-to-cell cross talk between HCC and immune cells (12). Exos are endosome-derived nanoscale (30–100 nm) lipid bilayer vesicles that contain several biological factors, including proteins, soluble substances, lipids, and miRNAs. They are transported to target cells to exert vital functions in intercellular cross talk. The characteristics and metabolism of immune cells can be modulated by tumor-derived Exos (13–17). Studies have shown that BAs regulate the immune microenvironment by stimulating the secretion of Exos from macrophages (18). In this study, we identified a new class of BAs and then tried to explore whether this BA class can affect the immune microenvironment of HCC by regulating Exos through FXR. Our study provides a new perspective for the protective effect of FXR in HCC patients. We recommend an FXR agonist combined with an anti-PD-1 antibody for immunotherapy of patients with advanced HCC.

## Materials and Methods

### Patient Samples and Cell Lines

HCC and liver tissues were obtained from the Third Affiliated Hospital of Sun Yat-sen University. All patients had not received antitumor therapy before surgery. The contents of 31 BAs were determined by analyzing the peritumoral liver tissues and tumor samples of 6 patients with HCC who underwent radical resection from June to September in 2019. In our study, each pair of analyzed tumor tissue and peritumoral liver tissue are from the same patient. Samples for immunohistochemistry (IHC) were collected from patients who underwent surgical resection in the same hospital from July 2010 to November 2011. The written informed consent was obtained from each participant. This study was approved by the Ethics Review Board of the Third Affiliated Hospital, Sun Yat-sen University. Humanized Huh-7 and LM3 cells and murine Hepa1-6 cells were obtained from ATCC.

### IHC Analysis

The tissues of HCC embedded in paraffin were cut into 4-µm thickness and IHC staining was performed as previously described (19). The following primary antibodies were used in follow-up experiments: SHP (sc-271511; Santa Cruz Biotechnology), FXR (ab129089; Abcam), and PD-L1 (13684S; CST). The staining results were independently analyzed by two pathologists who were blinded to the clinical outcomes. Because of their subcellular localization properties for normal functions, FXR and SHP in the nuclei and PD-L1 in the membranes of HCC cells were stained and scored for further analysis. The staining intensity of tumor cells was scored as 0 (negative), 1 (weak), 2 (moderate), 3 (high). The percentage of positive cells was categorized as follows: 0 (0%), 1 (1% to 25%), 2 (26% to 50%), 3 (51% to 75%), and 4 (76% to 100%). The total IHC staining score was obtained by multiplying the intensity score with the percentage score from 0 to 12. For FXR and SHP, staining scores 0–4 and 6–12 were considered as low and high expression, respectively. For PD-L1, staining scores 0–2 and 3–12 were defined as low and high expression, respectively (20).

### BAs Analysis

Ultra-performance liquid chromatography/tandem mass spectrometry (UPLC-MS/MS) was applied to measure the levels of BAs, namely, GLCA, 3-DHCA, LCA, 7-ketoLCA, NorCA, 7-DHCA, LCA S, HCA, UDCA, DCA, TLCA, CDCA-3Gln, TDCA, GDCA, GLCA-S, bUDCA, GHDCA, GHCA, CA, TwMCA, CDCA, TaMCA, THDCA, TLCA-S, THCA, TUDCA, GUDCA, TCA, GCA, GCDCA, and TCDCA, in the tumor and peritumoral liver tissue.

### Isolation of CD4^+^ or CD8^+^ T Cells and Flow Cytometry

Ficoll centrifugation (Axis-Shield) was used to isolate peripheral blood mononuclear from healthy donor blood samples. After 72 h of exposure to NorCA, LM3 cells or Exos were cocultured with CD4^+^ and CD8^+^ T cells that had been stimulated with anti-CD3/CD28 mAb beads. These results were analyzed by FlowJo 10.0 software. The fluorochrome-linked antibodies were applied as follow: anti-human Annexin V-FITC, PI-PE, CD4-APC-Cy7, CD8-FITC, PD-1-BV510, TIM3-PE, and CTLA4-PECY-Cy5.5 (eBioscience). Detailed staining protocols were followed previously described (21).

### Bicinchoninic Acid Assay

Bicinchoninic Acid Assay was used to detect protein concentration. The diluted protein was performed by BCA kit (ThermoFisher). The absorbance was detected at a wavelength of 562 nm.

### Quantitative Real-Time PCR (qRT-PCR)

Quantitative real-time PCR (qRT-PCR) was performed for mRNA detection using SYBR Green PCR Master Mix (Roche). The relative levels of mRNA were detected using the 2^-ΔΔCt^ method. Primer sequences are listed in **Supplementary Table 1**.

### Migration and Invasion Assays

To detect the wound healing ability of Huh-7 and LM3 cells, using a 200 μL pipette tip to scratch a straight wound, then observed and measured immediately and 24 hours after scratching. A 24-well transwell chambers coated with Matrigel (Corning Costar, Cambridge, MA, USA) was used to check cell invasion. The chamber has two culture compartments (upper and lower) separated by a polycarbonate membrane (Corning costar) with a pore diameter of 8 microns. The bottom chamber was filled with 600μL complete medium. 5 × 10^4^ per well cells were seeded in serum-free medium in the upper chamber. After culturing for 30 hours, cells that invaded to the bottom of the membrane were fixed with 4% paraformaldehyde and stained with 0.1% crystal violet, imaged, and counted under a microscope (Zeiss, Gottingen, Germany).

### Cell Proliferation Assay

For Cell Counting Kit-8 (CCK-8) assay (Dojindo, Kumamoto, Japan), transfected cells were seeded into 96-well plates at 1000 cells/well, and then, 10 µL of CCK-8 solution was added to each well and incubated for 4 h at 37°C. The absorbance was detected at a wavelength of 450 nm. For 5-ethynyl-2′-deoxyuridine (EdU) assay, transfected cells were seeded in 48-well plates. Next, the cells were stained using the Cell-Light EdU *In Vitro* Kit (RIBOBIO). Nuclei were stained with DAPI before being observed with fluorescence microscopy (Solarbio).

### Western Blot Analysis

Western blot analysis was employed as previously described (22). Anti-SHP (sc-271511; Santa Cruz Biotechnology), FXR (ab129089; Abcam), PD-L1 (13684S; CST), NSMase2 (ab68735; Abcam), RAB27A (ab55667; CST, USA), and β-Actin (ab8226; Abcam) were used according to concentration recommended by the manufacturers.

### Exos Isolation From Cell Lines

Exos were isolated from HCC cells and collected as described previously (23).

### Animal Studies

Male C57BL/6 and FXR-knockout mice aged 5 weeks were purchased from the Model Animal Research Center of Nanjing University (China) and Shanghai Nanfang Research Center for Model Organisms (China), respectively. All mice were raised under specific pathogen‐free (SPF) conditions. Before orthotopic implantation operation, the mice were deprived of water for 4 hours and food for 8 hours. 10% chloral hydrate (0.07 mL/10 g) was injected intraperitoneally for anesthesia. The mouse was disinfected with iodophor and fixed in supine position, and then a 1 cm opening was cut out 0.5 cm below the xiphoid process. Cutting the skin, peritoneum and muscle layer in order. The right liver lobe was exposed by slowly pressing the ribs. Hepa1-6 cells (1 × 10^6^ (50 μL)) were injected at an angle of 20° to the liver lobe. The injection extended for 1 cm and was performed slowly. After injection, the needle was pulled out and the hole was pressed for 1 minute until there was no active bleeding. Finally, the tissue was sealed layer by layer. After operation, water and food deprivation were performed for 4 hours. After 21 days, the liver tissues were harvested for detection. For the orthotopic implantation model, NorCA (5 mg/kg per mouse, Toronto Research Chemicals) and GW4064 (30 mg/kg per mouse, Sigma‐Aldrich) were intraperitoneally injected. To generate subcutaneous xenograft tumors, Hepa1-6 cells (1 × 10^6^) were suspended in 100 μL of phosphate-buffered saline and inoculated subcutaneously into the left flanks of mice. All mice were randomly divided into a control group and three treatment groups until the tumor volume reached 100 mm^3^. IgG2a was given to the control group, and the treatment groups were given intraperitoneal injection of anti-mouse PD-1 *InVivo*MAb (200 μg per mouse, Bio X Cell) every 3 days or GW4064 every day. For the subcutaneous xenograft model, NorCA was intratumorally injected. The microcaliper was used to measure the volume of the tumors twice per week. Tumor volume = (length × width^2^)/2. The animal research in this study were approved by the Institutional Animal Care and Use Committee of the Third Affiliated Hospital of Sun Yat-sen University (approval no 00256189).

### Lentiviral Vectors and Cell Infection

For stable knockdown of SHP and overexpression FXR, Hepa1-6 cells were seeded in 6-well plates (2.5 ×10^5^ cells) with antibiotic-free medium for 24 h. Then, they were infected with lentiviral hU6-SHP-ubiquitin-EGFP-IRES-puromycin and Ubi-FXR-CBh-gcGFP-IRES-puromycin or the corresponding control lentivirus (GeneChem Co., Ltd., Shanghai, China) at a multiplicity of infection (MOI) of 20 pfu/cell. The selection of stably transfected cells was performed 48 **h** later with 1 µg/mL puromycin (Sigma-Aldrich). The transfection efficiency of cherry fluorescent protein was observed by an inverted fluorescence microscope.

### Statistical Analysis

SPSS 17.0 (SPSS, Inc., Chicago, IL, USA) and Prism 6.0 (GraphPad Software, La Jolla, CA, USA) were used to analyze the data. The quantitative data were expressed as the means ± SD. Mann–Whitney *U* test, Student’s *t* test or Wilcoxon rank-sum test were used to compare two groups. χ2 test was used for correlation analysis. Kaplan-Meier survival analysis with log-rank test was performed to determine Overall survival (OS) and time-to-recurrence (TTR). The data were analyzed using two-sided test and *P* value of ≤ 0.05 was considered statistically significant in all analyses.

## Results

### UPLC–MS/MS Metabolomic Analysis

**Figure 1:**
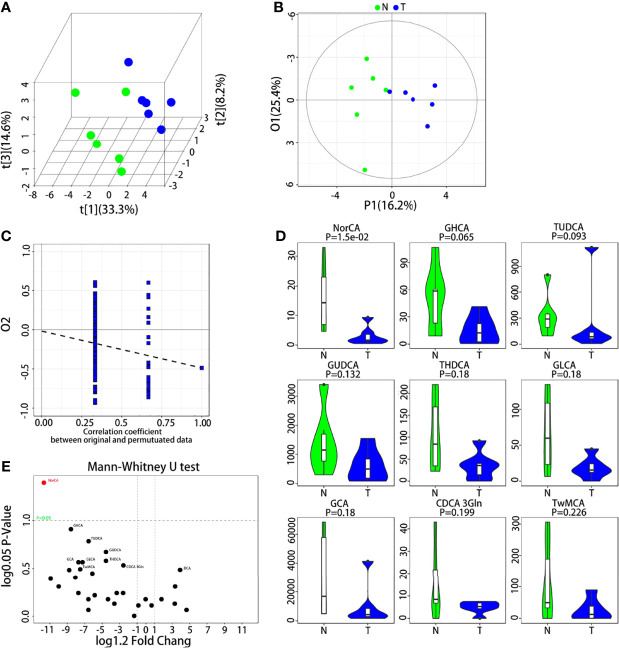
UPLC-MS/MS analysis of BAs in the tumor and peritumoral liver tissue. **(A, B)** Quantitative data of the BAs in the tumor and peritumoral liver tissue were analyzed with a PLS-DA plot and OPLS-DA plot, respectively. **(C)** Permutation test was applied to estimate the effectiveness of the advanced model. **(D)** The differential BAs were examined by Mann-Whitney U test for univariate statistical analysis, and we used violin diagrams to display the top 9 representative BAs. **(E)** All BAs obtained through the univariate statistical analysis are shown.

UPLC–MS was used to measure the concentrations of the 31 BAs in the analysis. In the subsequent multidimensional data screening process, PLS-DA (**Figure 1A**) and OPLS-DA (**Figure 1B**) patterns were used to show the aggregation trend of the tissue samples. The control group refers to 6 peritumoral liver tissues, and the model group refers to 6 tumor tissues paired with corresponding peritumoral tissues. The two groups showed obvious distinction, and these differences indicated that PLS-DA and OPLS-DA models were capable of distinguishing the tumor from the peritumoral liver tissue based on the BA levels. The permutation test was applied to estimate the effectiveness of the classification model. The intercept of Q2Y with a threshold less than zero indicates that this is a valid model (**Figure 1C**). The representative differential BAs were examined using the univariate statistical analysis Mann-Whitney U test (**Figure 1D**). The 31 differential BAs obtained by univariate statistical analysis are illustrated (**Figure 1E**). We found that NorCA was increased in peritumoral liver tissue compared to tumor tissue.

### NorCA Promotes the Migration and Invasion of HCC Cells

**Figure 2:**
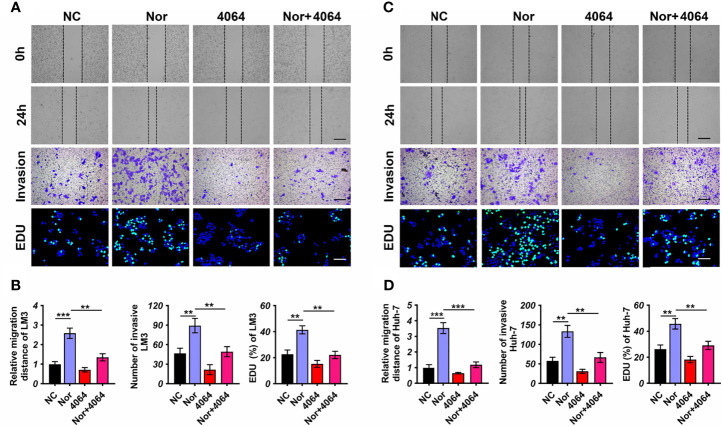
NorCA enhances the ability of HCC cells to migrate and invade and promotes cell proliferation. **(A-D)** Data showed that after NorCA exposure, the migration and invasion abilities of Huh-7 cells and LM3 cells were enhanced, while the FXR agonist GW4064 restored these trends (scale bar, 100 µm). EdU assay showed the consistent results (scale bar, 100 µm). **p < 0.01, and ***p < 0.001, n = 3. Cells treated with BSA were used as a negative control.

Although different types of BAs may have opposite effects in tumorigenesis (24, 25), the effect of NorCA on tumors is still unknown. In order to explore the influence of NorCA on HCC, we used NorCA to stimulate LM3 and Huh-7 cells and detected the migration and invasion of cells. To exclude the activation of these proteins through apoptosis induced by NorCA, we used flow cytometry and CCK-8 assays to detect the apoptosis of HCC cells after NorCA exposure. The toxicity of 200 µM NorCA was negligible in Huh-7 and LM3 cells (**Supplementary Figures 1A, B**). Our results showed that coculture with NorCA resulted in enhanced the migration (3.5- and 2.5-fold) and invasion (2.3- and 1.9-fold) abilities of the LM3 (**Figures 2A, B**) and Huh-7 (**Figures 2C, D**) cells. Based on a mass spectrometry analysis of the properties of the 31 BAs, a Pearson’s correlation analysis revealed that NorCA and GDCA were positively related (**Supplementary Figure 2**). The conjugated bile acid GDCA promoted the growth of tumors by downregulating the expression of FXR (26). Therefore, we hypothesized that NorCA can promote HCC migration and invasion by inhibiting FXR. GW4064 has been identified as an FXR agonist through experiments in which FXR was activated (27). As shown in **Figures 2A, B**, preincubation with while GW4064 efficiently prevented the HCC cells migration and invasion induced by NorCA. Meanwhile, in the cell proliferation assay, NorCA markedly promoted the proliferation of Huh-7 and LM3 cells and GW4064 restored these phenomena (**Figures 2A–D**).

### NorCA Upregulates PD-L1 Expression Through the FXR-SHP Axis

**Figure 3:**
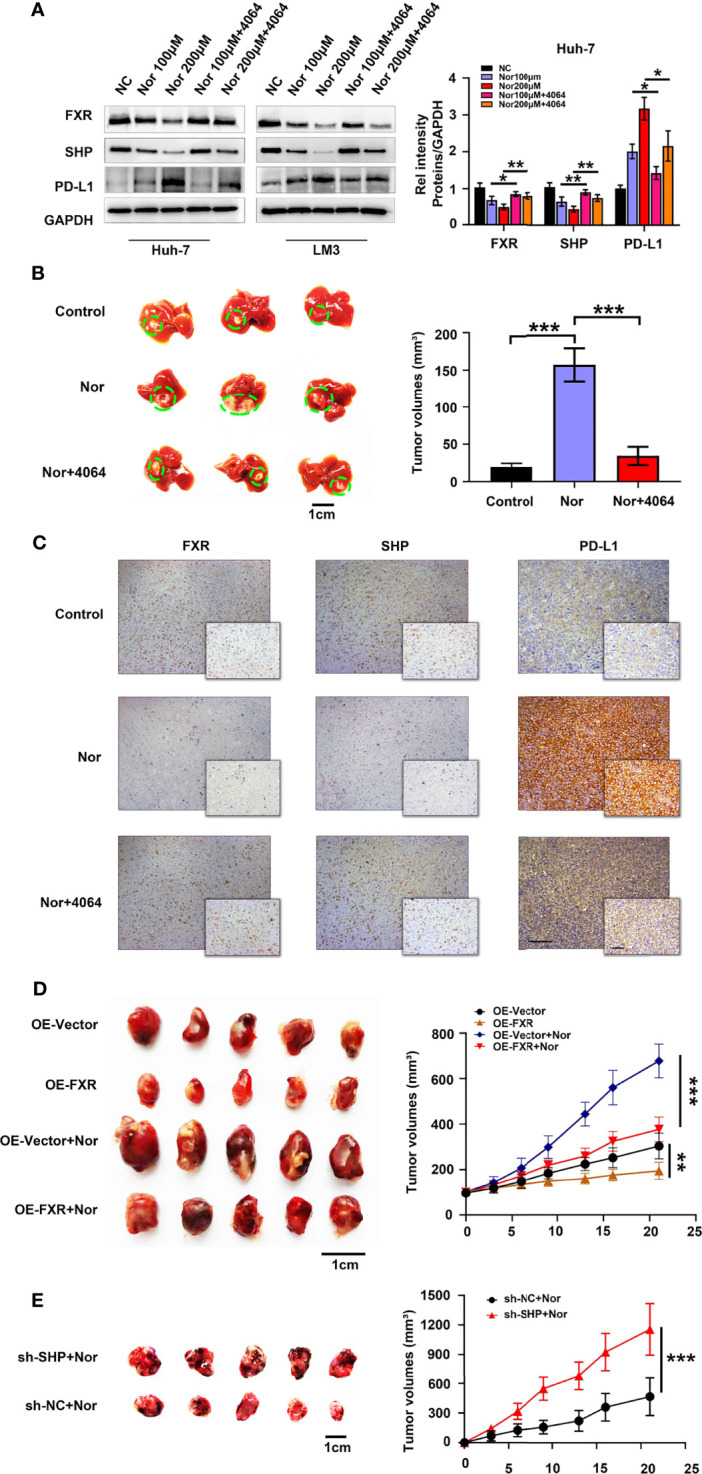
NorCA contributes to tumor growth and tumorigenicity *via* FXR-SHP-PD-L1 signaling. **(A)** Left graph, the FXR, SHP, and PD-L1 levels in Huh-7 and LM3 cells were detected by western blot analysis. Right graph, gray value analysis of all proteins. **(B)** Left graph, schematic representation of the orthotopic implantation formed by Hep1-6 cells. Mice treated with PBS (n=5), NorCA alone (n=5) or NorCA plus GW4064 (n=5). Right graph, quantification of tumor volumes in different groups. **(C)** Typical pictures of FXR, SHP and PD-L1 staining in orthotopic implantation models (Large figure: 200× magnification, scale bar, 100 µm. Small figure: 400× magnification, scale bar, 100 µm). **(D)** Left image, comparing the tumor volume of OE-FXR mice (n=7) with the tumor volume of OE-Vector (n=7) mice with or without NorCA treatment. Right graph, quantification of the tumor volumes in different groups. **(E)** Left graph, Hep1-6 cells with or without SHP knocked down were subcutaneously injected into mice. Tumor volumes in the SHP-knockdown group (n=5) was obviously higher than that in the control group (n=5). Right graph, quantification of tumor volumes of different groups. *p < 0.05, **p < 0.01, and ***p < 0.001.

To further explore how NorCA exerts its protumorigenic effect through FXR, we first determined the effect of NorCA on mRNA levels on downstream targets of FXR, including SHP, BSEP, ABCB4, CYP3A4, FGF19, and OSTα1. Our results indicated that the mRNA level of SHP was significantly downregulated by NorCA (**Supplementary Figure 3**). FXR is a nuclear receptor that plays vital role in the tumorigenicity of the liver by regulating SHP (28), and SHP can regulate the level of PD-L1 in cancer cells (20, 29). Consistent with these studies, Western blot showed that the NorCA-induced upregulation of PD-L1 expression involves the FXR/SHP signaling pathway, and preincubation with GW4064 efficiently reversed the decreased level of FXR and SHP and downregulated the expression of PD-L1 in a dose-dependent manner. (**Figure 3A** and **Supplementary Figure 4**). To verify the safety of the drug, we intraperitoneally injected mice with NorCA and then assessed liver and kidney function, performed blood tests and weighed the mice. We found that the toxicity of NorCA to C57BL/6 mice was negligible (**Supplementary Figure 5** and **Supplementary Table 2**). Moreover, our results showed that NorCA promoted tumor growth compared with the control group, and GW4064 significantly reduced tumor sizes in the treatment groups (**Figure 3B**). Consistently, HE staining also supports this result (**Supplementary Figure 6**). We further examined the expression of FXR, SHP and PD-L1 in liver tissue. Consistently, PD-L1 protein was significantly increased while FXR and SHP proteins were downregulated in the liver tissue of the NorCA group. Moreover, GW4064 treatment reversed these trends (**Figure 3C** and **Supplementary Figure 7**). Next, stable FXR overexpression (OE-FXR) in Hepa1-6 cells was utilized to explore the role of FXR in hepatocarcinogenesis induced by NorCA. FXR overexpression was confirmed by Western blot in Hepa1-6 (**Supplementary Figure 8**). The results showed that compared with control group, OE-FXR mice exhibited significantly decreased tumor size under NorCA stimulation (**Figure 3D**). Furthermore, stable SHP silencing in murine cancer cells was verified (**Supplementary Figure 9**), and our data indicated that knocking down SHP expression obviously promoted the tumorigenicity of Hepa1-6 cells induced by NorCA in mice (**Figure 3E**). In summary, these data demonstrate that NorCA drives tumor growth and tumorigenicity *via* FXR-SHP-PD-L1 signaling.

### NorCA Regulates Tumor-Derived Exos Through FXR to Affect the Immune Microenvironment

**Figure 4:**
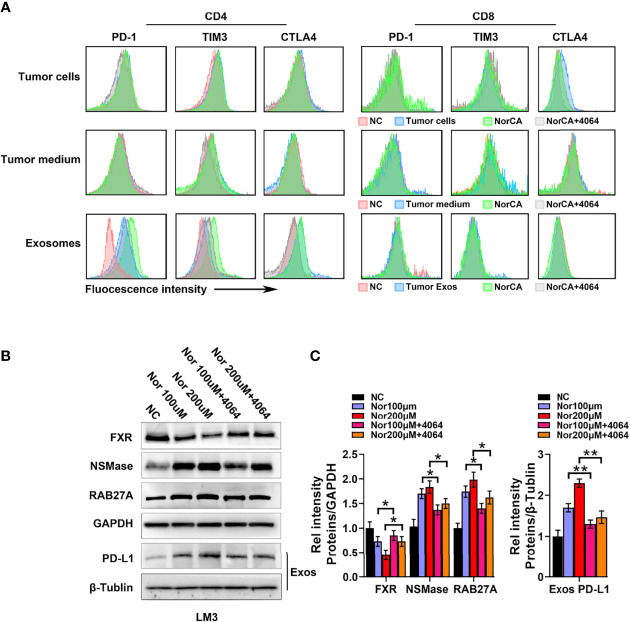
NorCA mediates the immune microenvironment by promoting tumor-derived Exos secretion. **(A)** After cells were exposed to NorCA, flow cytometry was applied to analyze the change in immune checkpoint-specific markers (PD-1, CTLA-4, and TIM3) in CD4^+^ T cells and CD8^+^ T cells cocultured with tumor cells, medium from tumor cell or tumor-derived Exos. Tumor-derived Exos regulated CD4^+^ T cells, as evidenced by the significantly higher expression of PD-1 and TIM3. **(B)** The FXR, NSMase, and RAB27A levels in tumor cells and PD-L1 levels in the tumor-derived Exos were detected using western blot analysis. **(C)** gray value analysis of all proteins. *p < 0.05 and **p < 0.01.

We further explored the impact of NorCA on the immune microenvironment. We characterized immune cell changes by flow cytometry using immune checkpoint-specific markers (PD-1, CTLA-4 and TIM3). However, we observed that the indicators of these immune checkpoints of CD4^+^ T cells and CD8^+^ T cells were not changed after coculture with LM3 cells induced by NorCA (**Figure 4A**). Moreover, we did not observe changes in these indicators when immune cells were cocultured in medium taken from cultures of LM3 cells induced by NorCA (**Figure 4A**). Deoxycholic acid can regulate macrophage-derived Exos to regulate the immune microenvironment (21). We hypothesized that NorCA-derived Exos (N-Exos) can play a role in modulating immune cells during tumor development. We found that N-Exos extracted from LM3 cells regulated CD4^+^ T cell protein expression, as evidenced by significantly higher expression of PD-1 and TIM3 but did not affect the expression of CTLA-4. Furthermore, N-Exos treated with GW4064 reversed these trends. However, these phenomena were not observed in CD8^+^ T cells (**Figure 4A**). To explore how NorCA affects tumor-derived Exos, we measured the protein expression of NSMase and RAB27A, which regulate the synthesis and secretion of Exos. Data showed that NorCA treatment increased the level of NSMase and RAB27A (**Figures 4B, C**). In addition, the total amount of exosomes in different treatment groups was detected by Bicinchoninic Acid Assay (BCA), the data was consistent with the previous results (**Supplementary Figure 10**). Furthermore, NorCA also upregulated PD-L1 expression on Exos secreted from HCC cells (**Figures 4B, C**). Moreover, GW4064 reversed the increased generation and secretion of Exos and the increased level of PD-L1. The results suggest that NorCA may create strong immunosuppressive microenvironment to promote the immune escape of HCC cells.

### Upregulation of PD-L1 Level by FXR Is Used to Stratify HCC Patients

**Figure 5:**
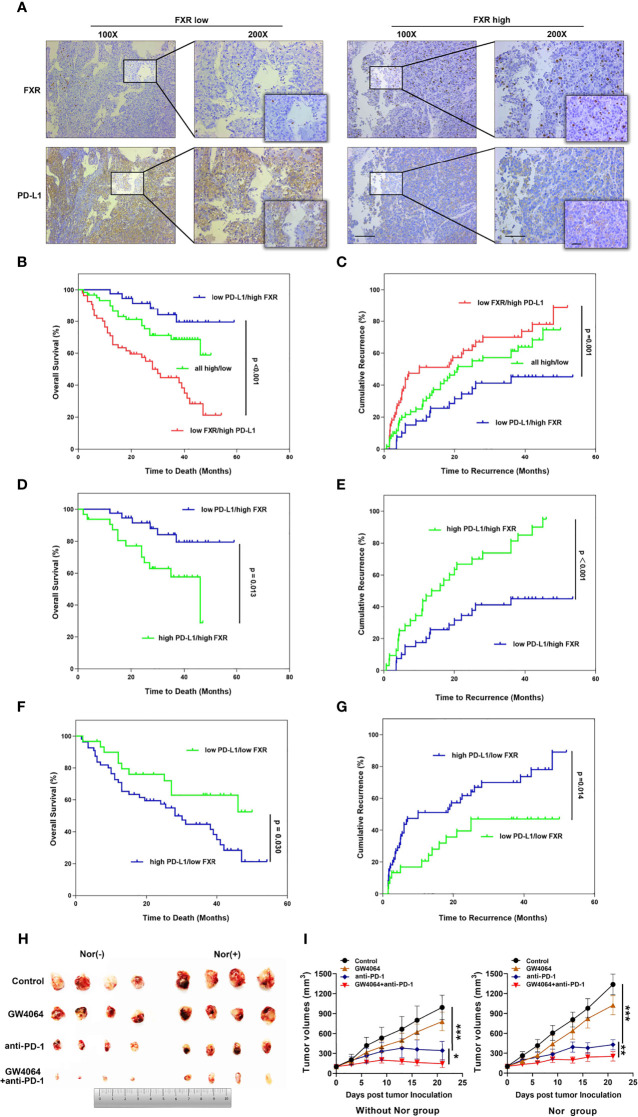
The relevance of FXR to PD-L1 level in HCC samples and the FXR agonist exerting synergistic effects with anti-PD-1 Ab in the mouse model. **(A)** Immunohistochemical staining was performed with 156 HCC tissues. Representative images of FXR^low^PD-L1^high^ staining and FXR^high^PD-L1^low^ staining of serial sections of HCC tissues are displayed at 100× (left graph), 200× (right graph), 400× (right small graph) magnifications (scale bar, 100 µm). **(B, C)** The OS and TTR of postoperative HCC patients based on both FXR and PD-L1 expression. Patients with FXR^low^PD-L1^high^ displayed the shortest OS (p<0.001, log-rank test) and TTR (p=0.001, log-rank test). **(D–G)** The OS and TTR for 4 subgroups (FXR^high^PD-L1^high^ VS FXR^high^PD-L1^low^, and FXR^low^PD-L1^high^ and FXR^low^PD-L1^low^). **(H)** Mice were inoculated subcutaneously with 1 × 10^6^ Hep1-6 cells and treated with anti-PD-1 Ab, IgG2a, GW4064 or anti-PD-1+GW4064 after the tumors reached 100 mm^3^. **(I)** With (right graph) or without (left graph) NorCA exposure, the tumor growth in the single-agent therapy group (n = 5) was compared with the combination therapy group (n = 5). *p < 0.05, **p < 0.01, and ***p < 0.001.

Taken together, these data showed that FXR can regulate PD-L1 through transrepression and SHP signaling in HCC cells. Considering this foundation, we first sought to explore the relationship between FXR and PD-L1 *in vivo*. A total of 156 HCC specimen cohorts were used to estimate PD-L1 and FXR expression by IHC staining. Interestingly, the intensity of the PD-L1 staining was distinctly higher in “FXR low” samples than in “FXR high” samples (**Figure 5A**). The spearman correlation analysis indicated a statistically significant negative correlation between PD-L1 and FXR in the HCC tissues (**Supplementary Figure 11**). We found that the proportion of FXR^low^PD-L1^high^ subgroup was 35% (55/156) in the HCC samples. OS is defined as the time from the end of the first operation to death (for any reason). TTR is defined as the time from the end of the first operation to the first recurrence. The OS and TTR for all 3 subgroups (FXR^low^PD-L1^high^, FXR^high^PD-L1^low^, and FXR^high^PD-L1^high^ and FXR^low^PD-L1^low^) are presented in **Figures 5B, C**. Here, we found that the FXR^low^PD-L1^high^ subgroup had a significantly shorter OS (p<0.001) and TTR (p=0.001) than the FXR^high^PD-L1^low^ HCC groups. In order to further isolate the potential effect of the relationship between FXR and PD-L1, we next compare identical levels of FXR with different levels of PD-L1. The data showed that whether in the FXRhigh or FXRlow group, the PD-L1low subgroup showed better OS and TTR than the PD-L1high expression group (**Figures 5D–G**). These data indicated that PD-L1 is a prognostic factor independent of FXR.

### FXR Agonist Combined With Anti-PD-1 Ab in the HCC Syngeneic Mouse Model

Because NorCA showed an immunomodulatory effect in CD4^+^ T cells, we hypothesized that an FXR agonist in combination with an immune checkpoint inhibitor may have synergistic antitumor effect. Thus, we detected the antitumor therapeutic ability of GW4064 combined with anti‐PD‐1 Ab in the mouse model. In the group with or without NorCA, compared with the nontreatment group, GW4064 impeded tumor growth while the anti‐PD‐1 Ab significantly depressed tumor progress (**Figure 5H**). GW4064 combined with anti‐PD‐1 Ab treatment led to tumor regression and exhibited the most effective antitumor ability (**Figure 5I**). Thus, GW4064 combined with anti‐PD‐1 Ab treatment exhibited potent antitumor capacity because of the immune‐activating efficacy of anti‐PD‐1 Ab.

## Discussion

Here, we identified a previously unrecognized subset of protumorigenic bile acid and employed various analytic strategies to assess the biological effects and mechanisms of this bile acid *in vivo* and *in vitro*. Our research illustrated that the FXR-SHP-PD-L1 axis may be a new way for NorCA to promote the tumorigenesis of HCC cells. Additionally, NorCA can increase the secretion of Exos from HCC cells, and N-Exos play roles in regulating CD4^+^ cells during tumor progression. Specifically, compared with either therapy administered alone to the HCC tumor-bearing syngeneic model mice, a combination treatment consisting of FXR agonist plus an anti‐PD‐1 Ab obviously inhibited tumor development and showed potent antitumor ability.

Recently, BAs have been recognized as pivotal contributors to the etiopathogenesis of gastroenteric disorder and tumors. According to the report, BAs were enriched in cancer patients and were associated with poor prognosis (30). However, different BAs that exist in tumor microenvironments exhibit distinct efficiencies and functions (31, 32). Important secondary BAs, such as TCDCA, GCA, GCDCA, and GDCA, have all been revealed as etiologic agents in gastrointestinal tumors (33). Consistent with these studies, we illustrated that in the context of HCC, NorCA, a previously unrecognized BA subset, exhibited protumorigenic properties in the present study. FXR has been demonstrated to be a regulatory element of immune responses in different diseases in addition to its role in modulating BA metabolism (34, 35). However, the correlation between the immune microenvironment and FXR in HCC remains poorly understood. Here, we demonstrated that FXR decreases the level of PD-L1 in HCC cells under NorCA exposure. Exos have emerged as vital contributors in HCC etiopathogenesis, and the list of indicated tumor-derived Exos that regulate immunomodulatory effects is increasing (36, 37). However, few studies have focused on FXR and Exos, and the potential interaction between them remains largely unknown. Metastatic melanoma releases high levels of extracellular vesicles, mainly in the form of exosomes, carrying PD-L1 on its surface (38). Interestingly, our results indicated that FXR can impede the generation and secretion of tumor Exos, and an FXR agonist can inhibit the increased expression of PD-L1 on N-Exos while restoring the modulation of CD4^+^ T cells by N-Exos. Therefore, we have identified a new function of FXR, which exerts pivotal effect in the immune microenvironment, not only by regulating the level of PD-L1 in HCC cells but also by affecting tumor-derived Exos to regulate CD4^+^ T cell immune costimulatory targets.

Given that NorCA can exert an immunosuppressive effect by regulating PD-L1 through FXR in HCC cells, we further explored the relevance of FXR to PD-L1 *in vivo*. Moreover, a negative correlation between PD-L1 and FXR level was observed in 156 HCC patients. We identified the FXR^low^PD-L1^high^ and FXR^high^PD-L1^low^HCC subgroups. FXR clearly downregulated PD-L1 in HCC cells, and the FXR^high^PD-L1^low^ subgroup was associated with a better outcome for HCC patients. Our data indicate that this relevance may be due to the high expression of FXR in HCC cells, which inhibits PD-L1 level and thereby acting as a protective contributor against cancer progress. On the contrary, the FXR^low^PD-L1^high^ subgroup was correlated with a poor prognosis. In particular, immune checkpoint inhibitors represented by PD-L1/PD-1-blocking antibodies have obvious curative effect on the treatment of patients with advanced HCC (39). However, not all patients show a complete response or benefit from anti-PD-1/PD-L1 therapy. Because of the complexity of immunomodulatory mechanisms and the heterogeneity of tumors, combination therapy is a promising clinical treatment that can overcome the limitations of single-agent therapy (40). In agreement with this notion, our experiments indicated that combination treatment with an FXR agonist plus an anti‐PD‐1 Ab shows preferable antitumor ability in model mice.

There are several limitations in our study. First, evidence have demonstrated that other types of BAs can also increase or decrease FXR expression and participate in tumor progression (41). Therefore, we should have explored the effect of NorCA treatment on the concentrations and composition of other BAs, so as to determine whether NorCA regulates FXR signaling *via* multiple mechanisms. Second, we did not clarify whether OE-FXR or sh-SHP could affect the level of endogenous NorCA in tumor-bearing mouse models. If OE-FXR or sh-SHP really affected the endogenous NorCA level *in vivo*, the effect of NorCA treatment on tumor growth may be overestimated or underestimated. Last but not least, FXR has been uncovered to regulate the production of various inflammatory cytokines (42). Furthermore, a large number of studies have indicated that some cytokines play a critical role in tumor immune escape (43, 44). Hence, it would be very interesting to explore whether NorCA promotes tumor immune escape by inducing the secretion of specific inflammatory cytokines.

**Figure 6:**
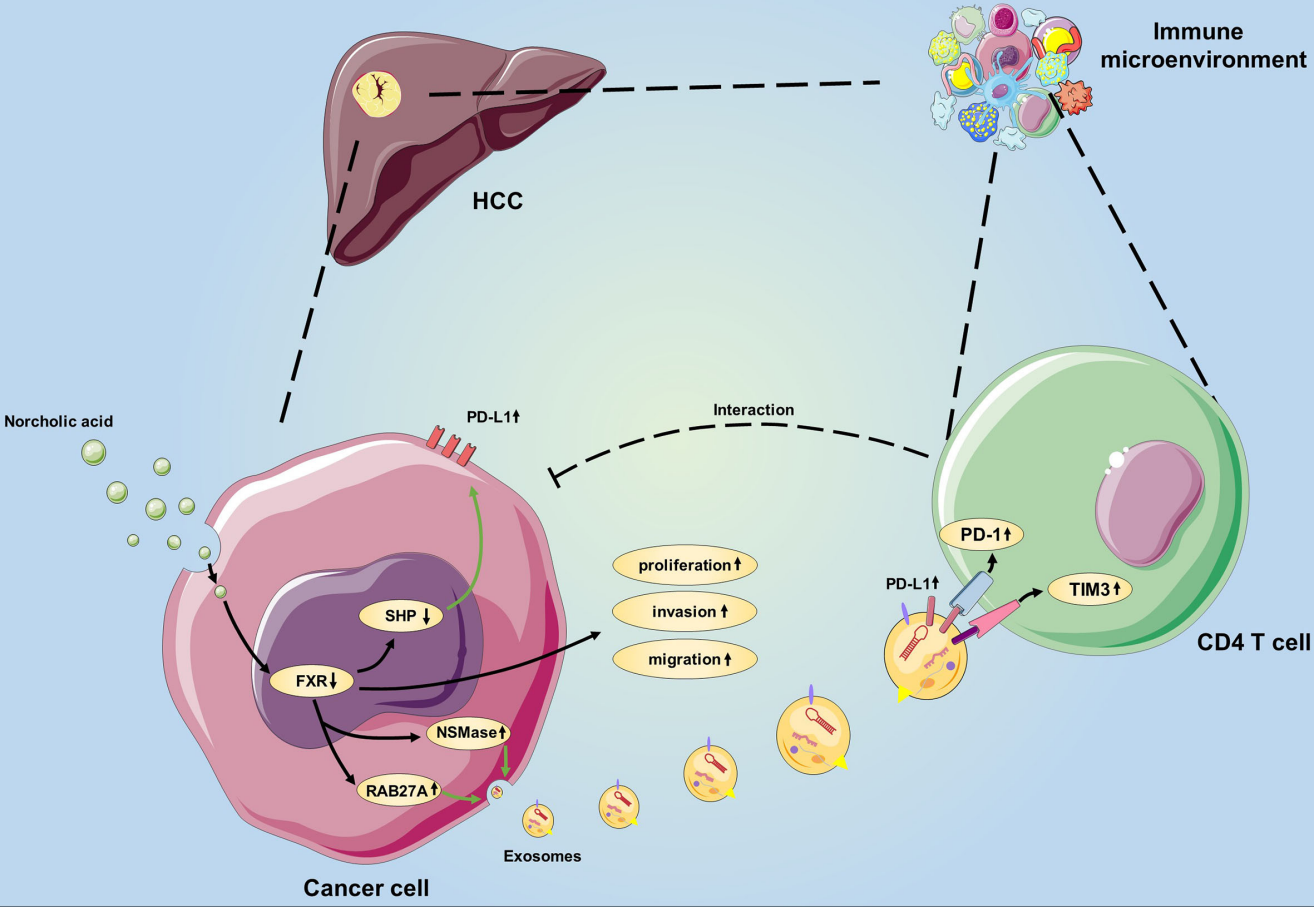
Schematic showing the novel bile acid-mediated immune microenvironment in HCC progression. Considering our study results, we propose a model involving NorCA in HCC development. First, NorCA interacts with HCC cells to promote their ability to express PD-L1 through the FXR-SHP pathway. Second, NorCA enhances the synthesis and secretion of Exos from HCC cells by regulating NSMase and RAB27A. Third, N-Exos have the potential to create an immunosuppressive microenvironment by regulating the function of CD4^+^ T cell, thus creates beneficial conditions for tumor development.

Herein, we for the first time demonstrate that NorCA can enhance HCC cell proliferation, migration and invasion by negatively regulating FXR. In addition, NorCA can increase PD-L1 levels on the surfaces of HCC cells and their exosomes, and NorCA-induced exosomes significantly impair the function of CD4^+^ T cells. Furthermore, FXR agonist can synergize with anti-PD-1 Ab to inhibit HCC growth *in vivo*. Taken together, these results suggest that NorCA can facilitate HCC progression and tumor escape (**Figure 6**), and the combination of anti-PD-1 Ab and FXR agonist may be a promising strategy to combat HCC. However, more well-designed animal and clinical trials are warranted to further validate our findings in future due to the limitations in our study.

## Data Availability Statement

The original contributions presented in the study are included in the article/**Supplementary Material**. Further inquiries can be directed to the corresponding authors.

## Ethics Statement

The studies involving human participants were reviewed and approved by the Institutional Animal Care and Use Committee of the Third Affiliated Hospital of Sun Yat-sen University. The patients/participants provided their written informed consent to participate in this study. The animal study was reviewed and approved by the Institutional Animal Care and Use Committee of the Third Affiliated Hospital of Sun Yat-sen University.

## Author Contributions

WL, LY, and YY was the principal investigator and designed the research. YG, KL, YQ, KZ, JL, and SH performed the experiments. YG, KL, YQ, YC, and HY analyzed the results and wrote the manuscript. All authors contributed to the article and approved the submitted version.

## Funding

This work was supported by: National 13th Five-Year Science and Technology Plan Major Projects of China (2017ZX10203205); National Key R&D Plan (2017YFA0104304); National Natural Science Foundation of China (81770648, 81972286, 81970509); Guangdong Natural Science Foundation (2018A030313259, 2015A030312013); Science and Technology Program of Guangdong Province (2017B020209004, 20169013, 2020B1212060019); Science and Technology Program of Guangzhou city (201508020262); Guangdong Basic and Applied Basic Research Foundation (2019A1515110654); The Fundamental Research Funds for the Central Universities (20ykpy38); China Postdoctoral Science Foundation (2019TQ0369, 2020M672987).

## Conflict of Interest

The authors declare that the research was conducted in the absence of any commercial or financial relationships that could be construed as a potential conflict of interest.

## Publisher’s Note

All claims expressed in this article are solely those of the authors and do not necessarily represent those of their affiliated organizations, or those of the publisher, the editors and the reviewers. Any product that may be evaluated in this article, or claim that may be made by its manufacturer, is not guaranteed or endorsed by the publisher.
