# Supplementary material for: Norcholic Acid Promotes Tumor Progression and Immune Escape by Regulating Farnesoid X Receptor in Hepatocellular Carcinoma
Source: Front Oncol. 2021 Nov 23;11:711448. doi: 10.3389/fonc.2021.711448 (PMC8648605; doi:10.3389/fonc.2021.711448)
Supplement: Supplementary file 1 [file DataSheet_1.docx]

Supplementary Material

**Supplementary Figure 1**


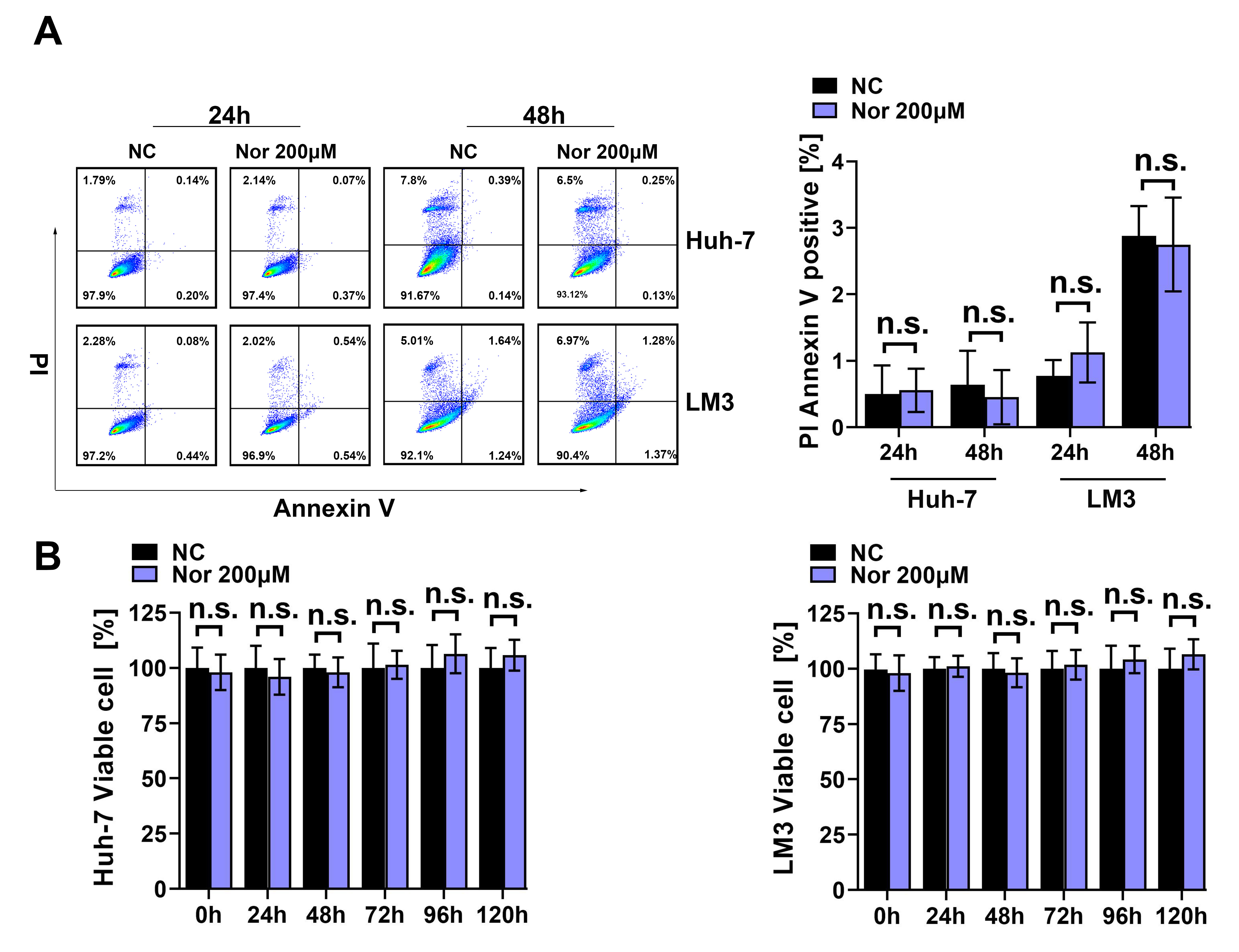


**Legend Supplementary Figure 1**

NorCA toxicity was detected by flow cytometry and CCK-8 assay in HCC cells. **(A)** Left graph, using Annexin V-FITC/PI double staining to detect the apoptosis of Huh-7 and LM3 cells. Right graph, quantification of apoptosis rate of NorCA on cells. **(B)** CCK-8 assay was applied to measure cell proliferation. Huh-7 and LM3 cells were exposed to 200 µM NorCA for different durations. Data are presented as the mean ± SD. n.s., not significant, *p<0.05, **p<0.01, and ***p<0.001.

**Supplementary Figure 2**


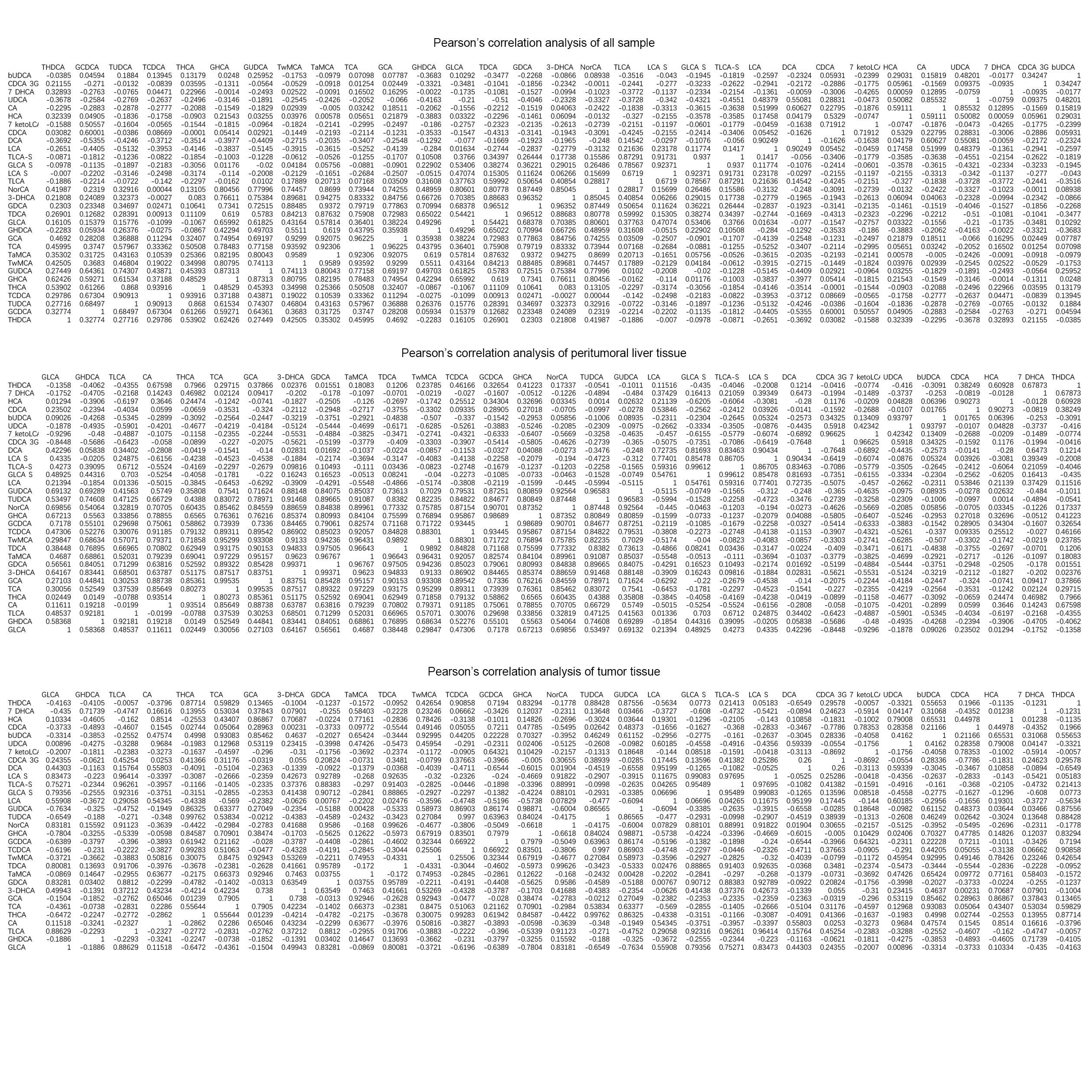


**Legend Supplementary Figure 2**

Pearson's correlation analysis of 31 BAs in all specimens, and separately analyzed the correlation of BAs in tumor tissues and peritumoral liver tissues.

**Supplementary Figure 3**


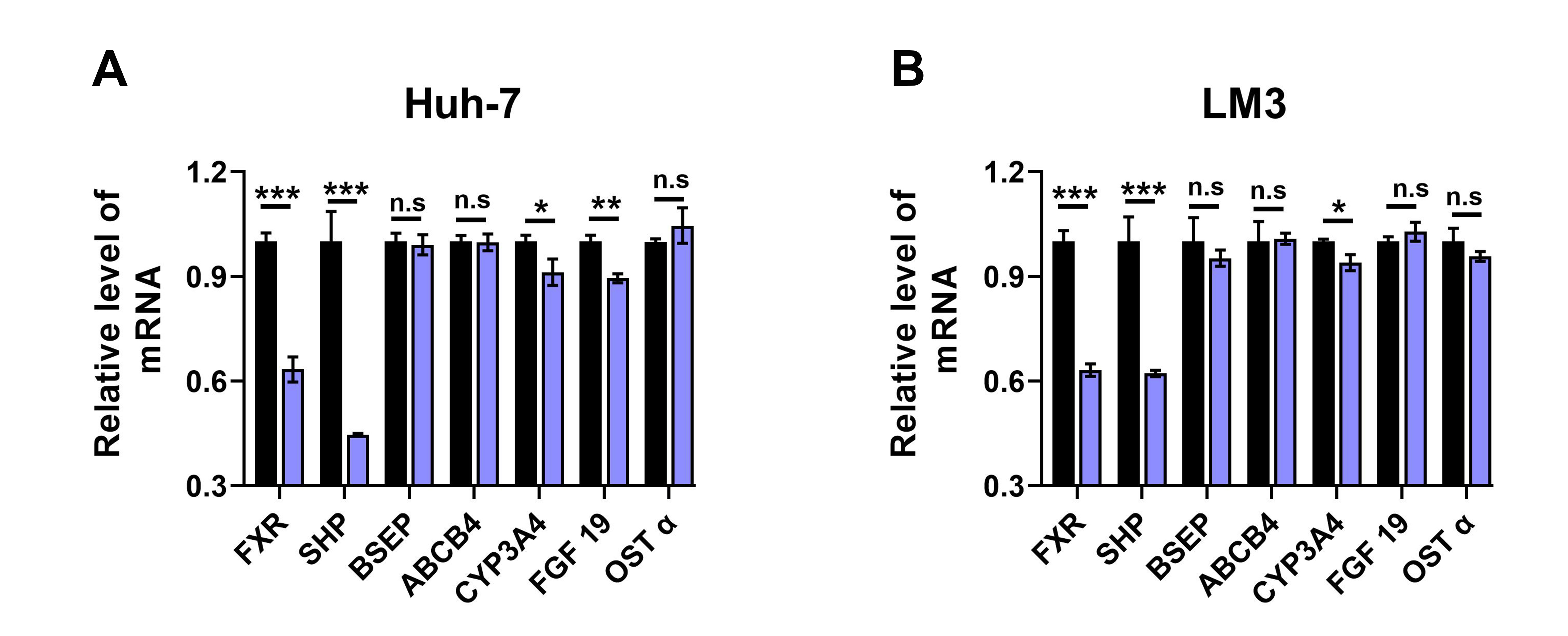


**Legend Supplementary Figure 3**

mRNA levels on downstream targets of FXR, including SHP, BSEP, ABCB4, CYP3A4, FGF19, and OSTα1. Data are presented as the means ± SD. n.s., not significant, *p<0.05, **p<0.01, and ***p<0.001.

**Supplementary Figure 4**


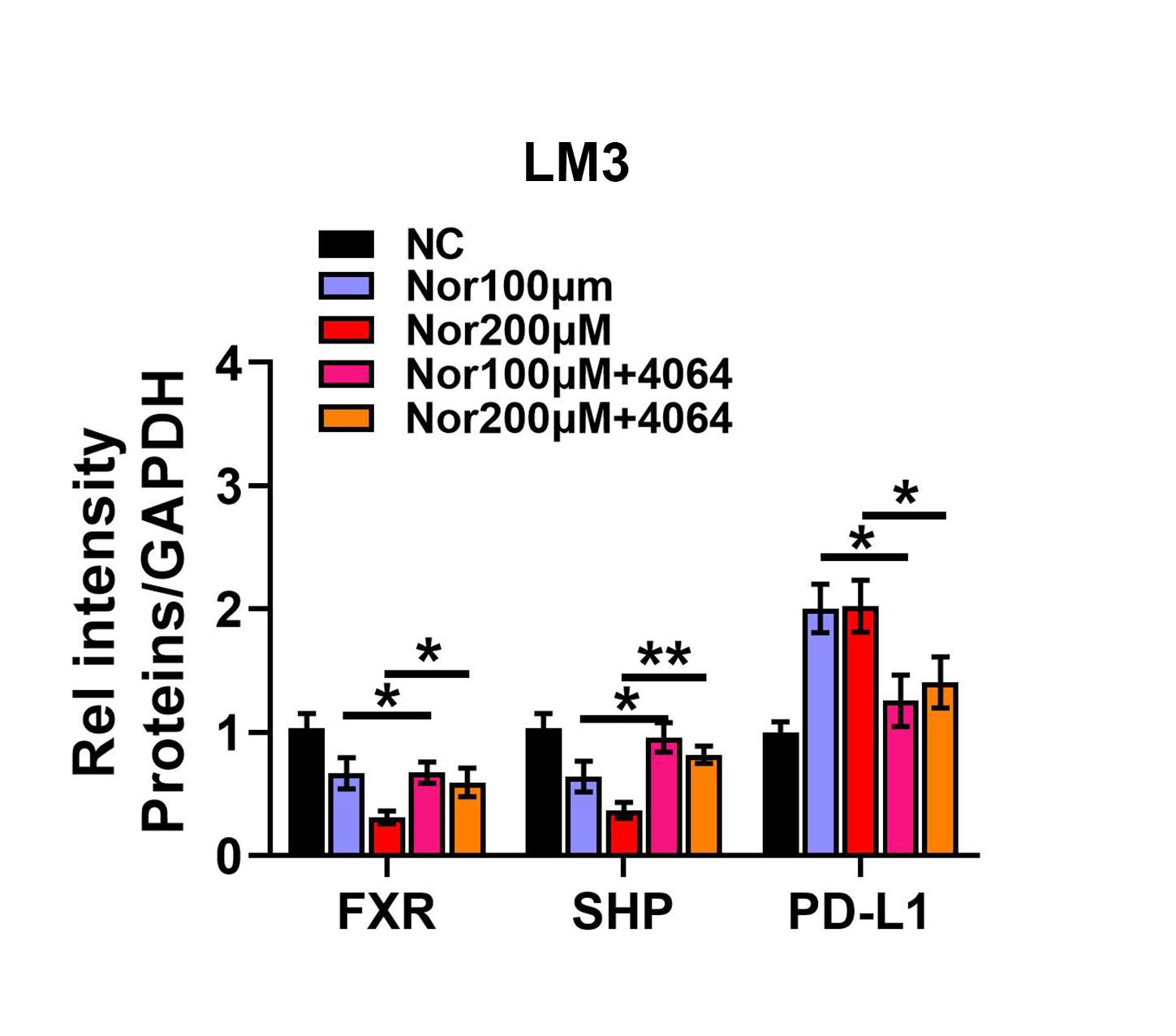


**Legend Supplementary Figure 4**

Gray value analysis of all proteins in LM3 cells. n.s., not significant, *p<0.05, **p<0.01, and ***p<0.001.

**Supplementary Figure 5**


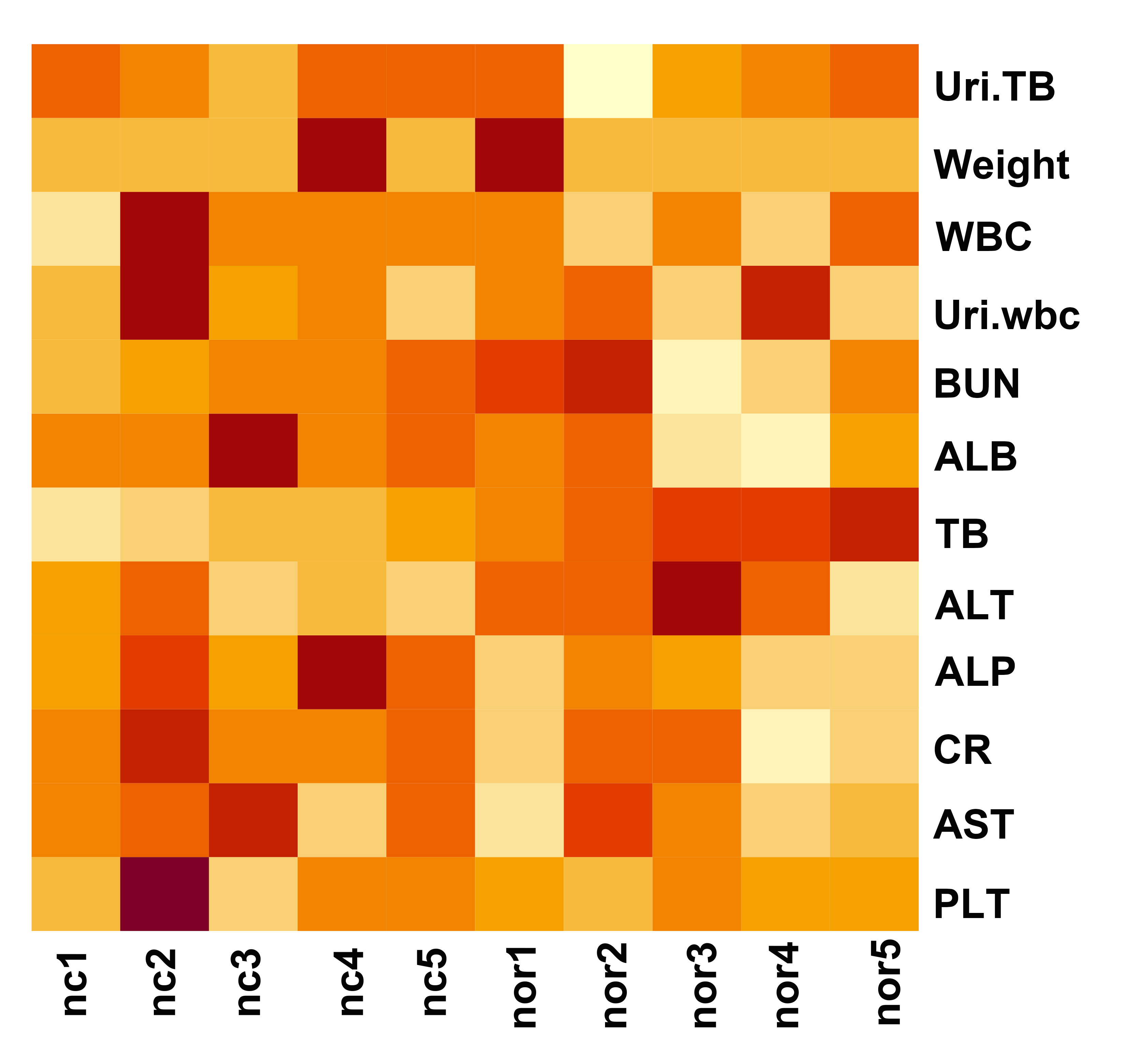


**Legend Supplementary Figure 5**

NorCA toxicity was detected in mice. After intraperitoneal injection of NorCA for 21 days, we detected liver function (AST, ALT, ALP, ALB, and TB levels), kidney function (BUN and CR levels), blood (WBC and PLT count), urine (Uri.TB level and Uri.wbc) and weight of the mice. The results are exhibited in a heat map. Compared with that of the control group (n=5), the toxicity of the NorCA group (n=5) was negligible.

**Supplementary Figure 6**


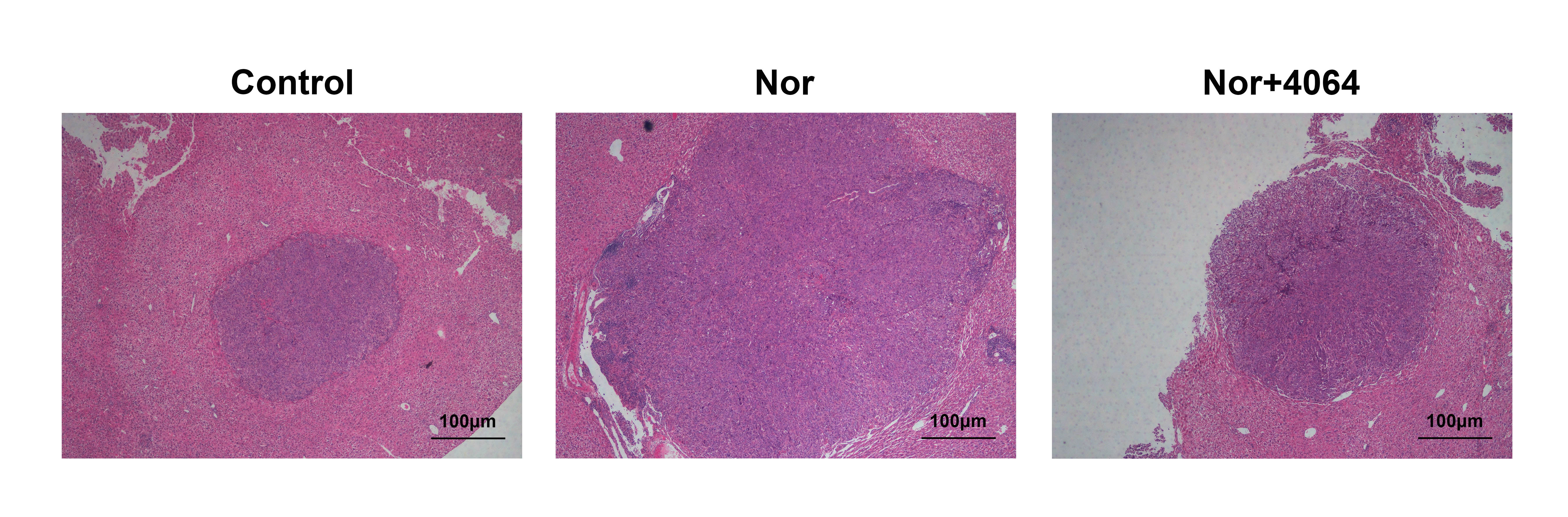


**Legend Supplementary Figure 6**

HE staining of different treatment of mice group.

**Supplementary Figure 7**


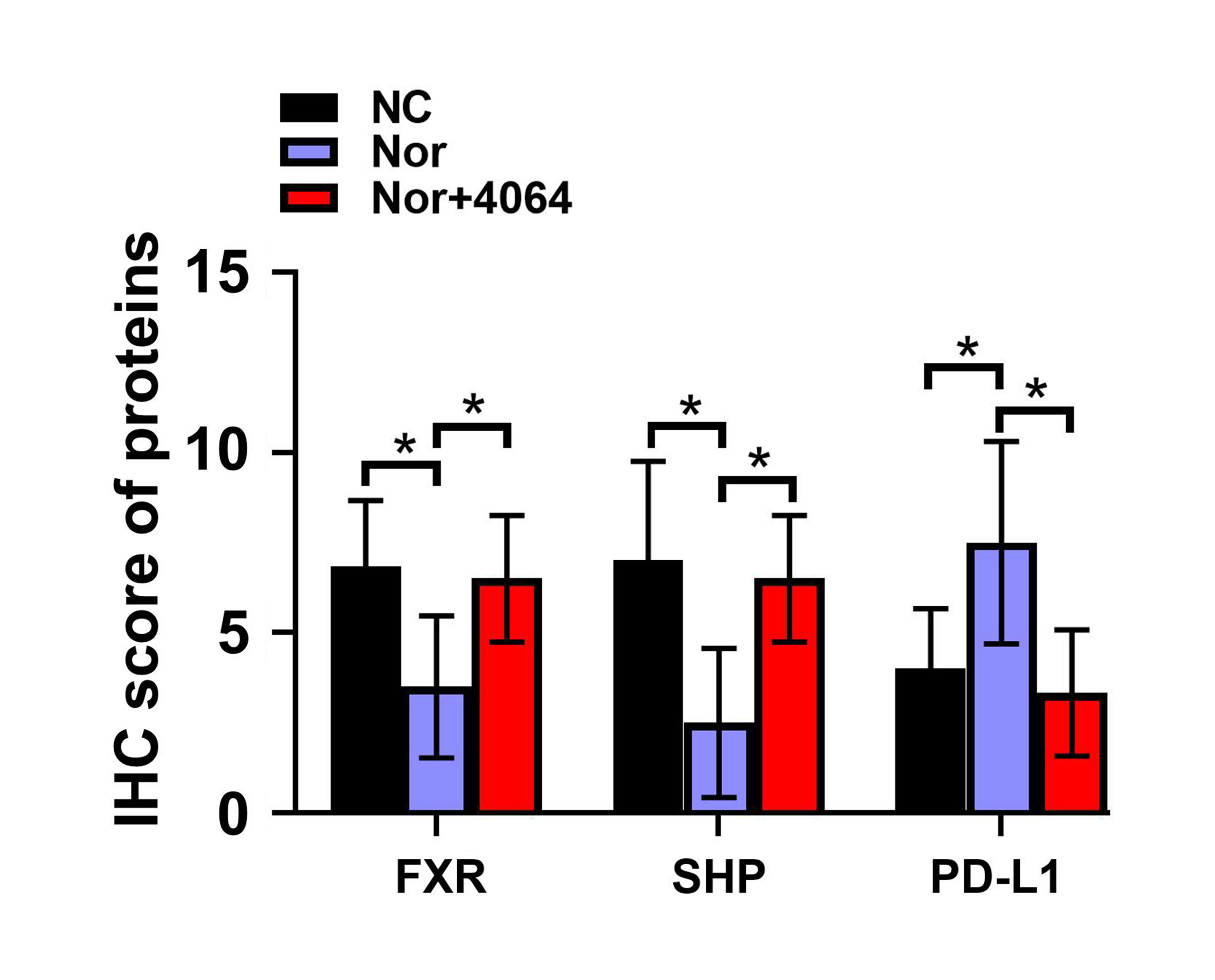


**Legend Supplementary Figure 7**

The IHC score of various proteins. The FXR, SHP and PD-L1 scores in different groups are illustrated (Mann–Whitney U test). Data are presented as the means ± SD. n.s., not significant, *p<0.05, **p<0.01, and ***p<0.001.

**Supplementary Figure 8**


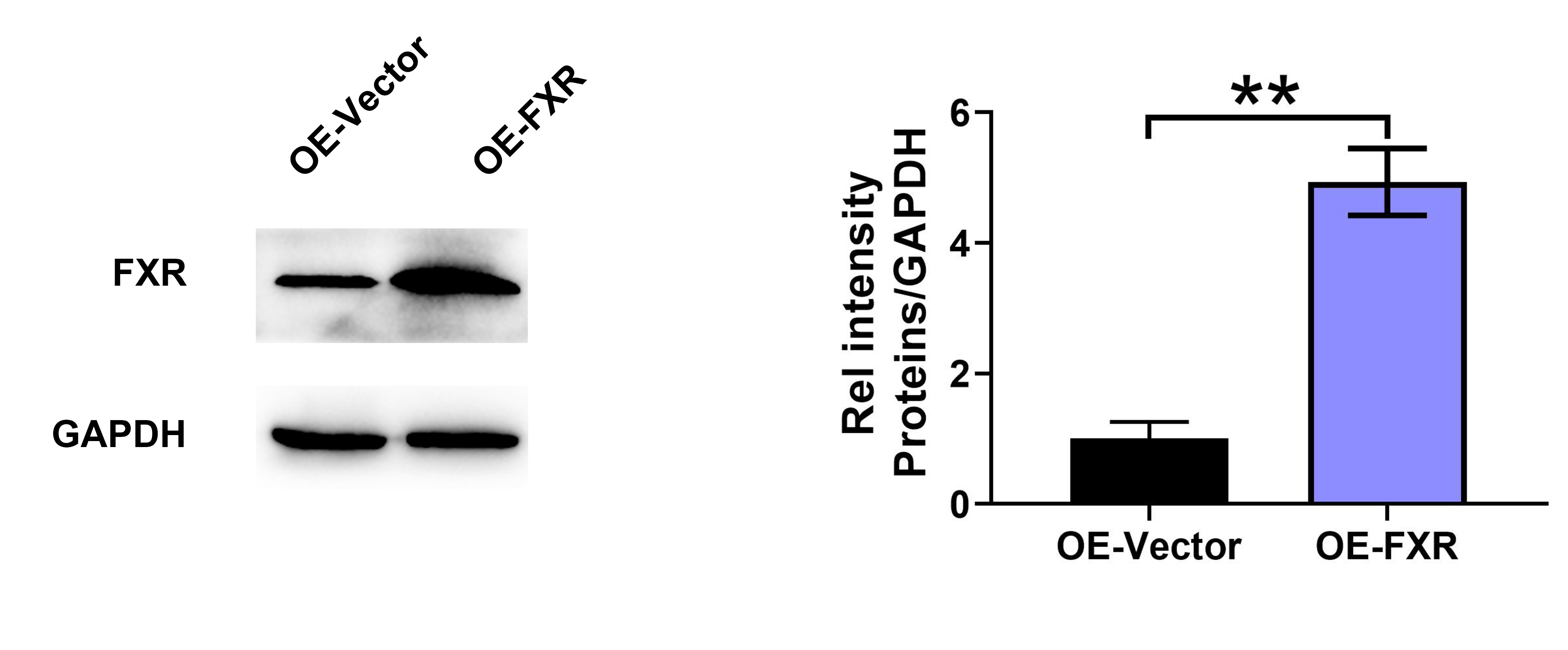


**Legend Supplementary Figure 8**

Left graph, the overexpression of FXR level in Hepa1-6 cells was measured by western blot analysis Right graph, gray value analysis of the proteins. Data are presented as the mean ± SD. n.s., not significant, *p<0.05, **p<0.01, and ***p<0.001.

**Supplementary Figure 9**


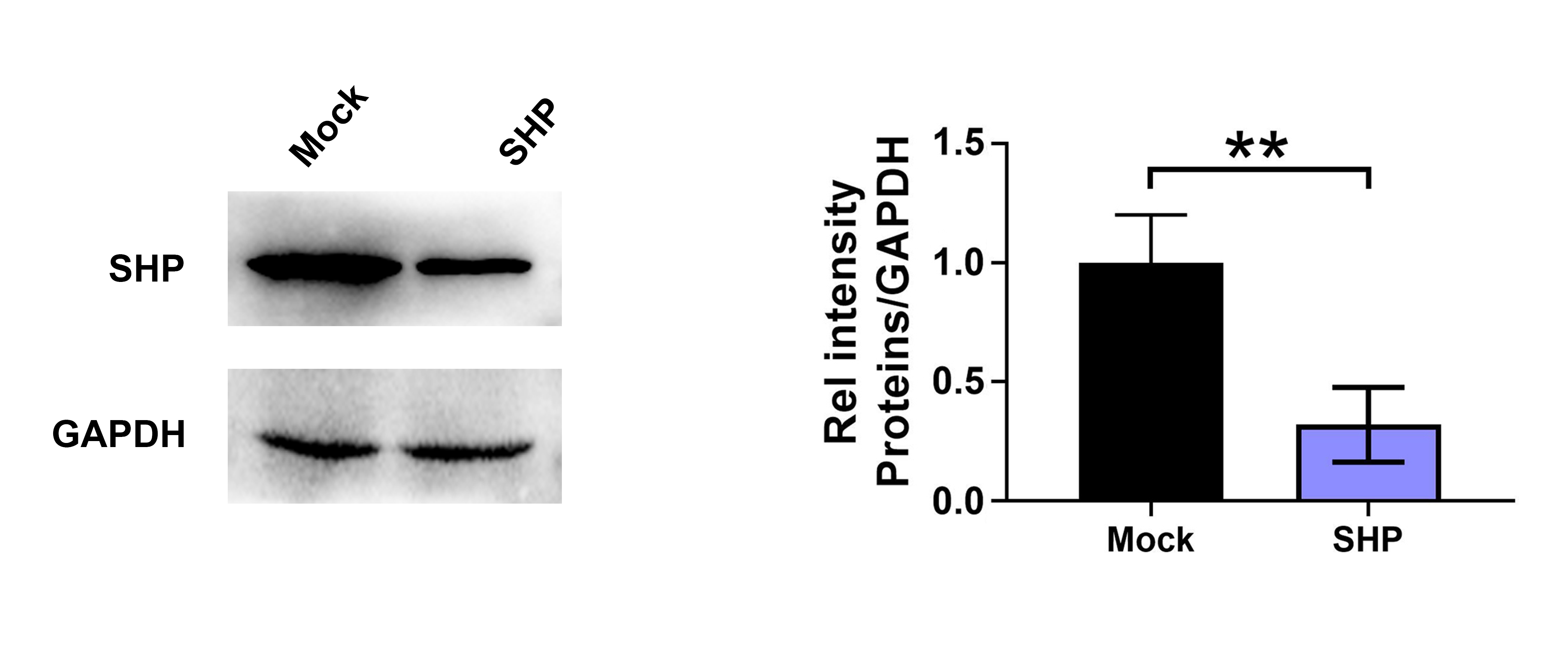


**Legend Supplementary Figure 9**

Left graph, after infection with lentiviral vectors, the SHP level in Hepa1-6 cells was measured by western blot analysis Right graph, gray value analysis of the proteins. Data are presented as the mean ± SD. n.s., not significant, *p<0.05, **p<0.01, and ***p<0.001.

**Supplementary Figure 10**


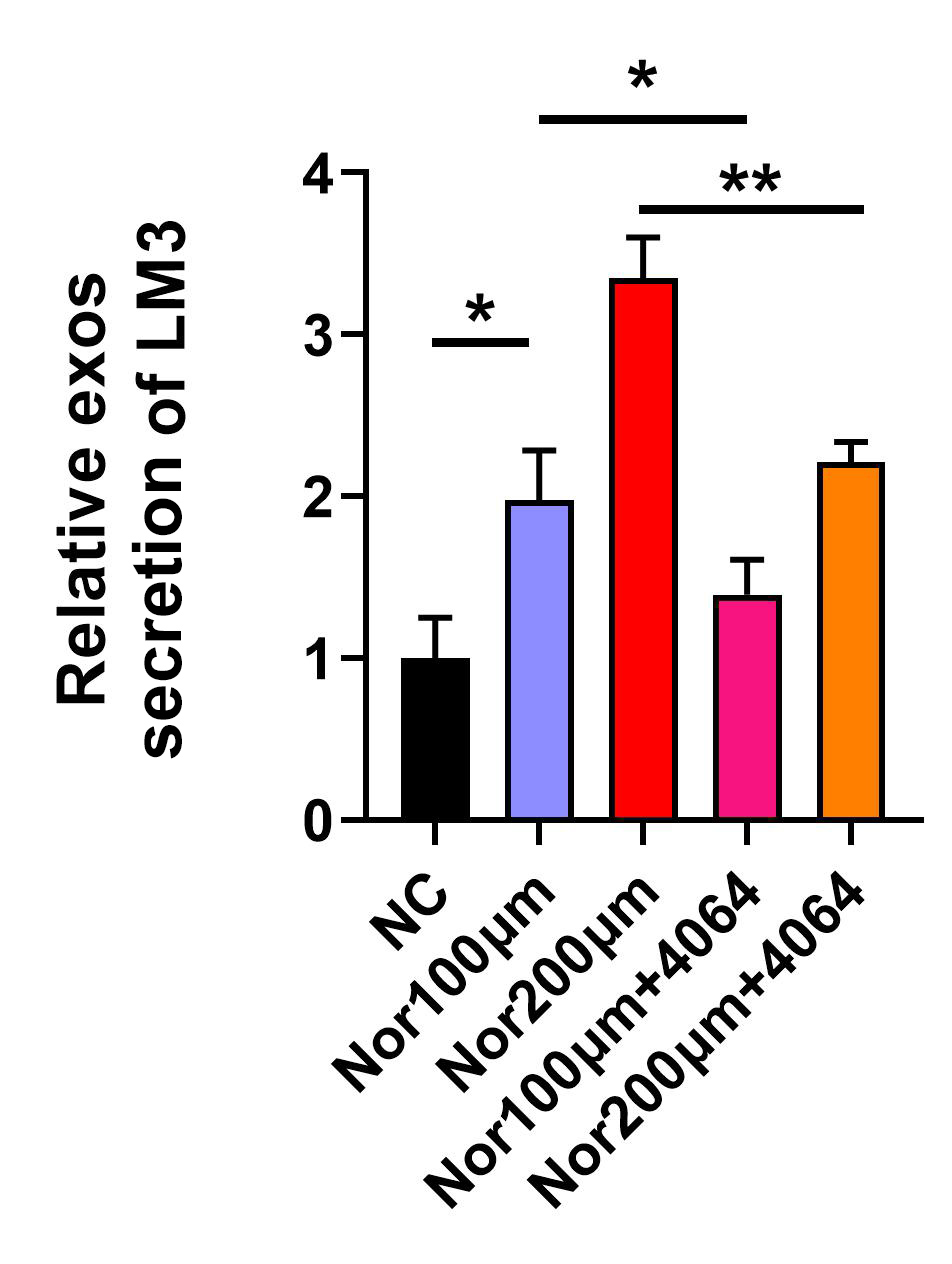


**Legend Supplementary Figure 10**

Relative exosomes secreted by LM3 cells in different treatment group. Data are presented as the mean ± SD. n.s., not significant, *p<0.05, **p<0.01, and ***p<0.001.

**Supplementary Figure 11**


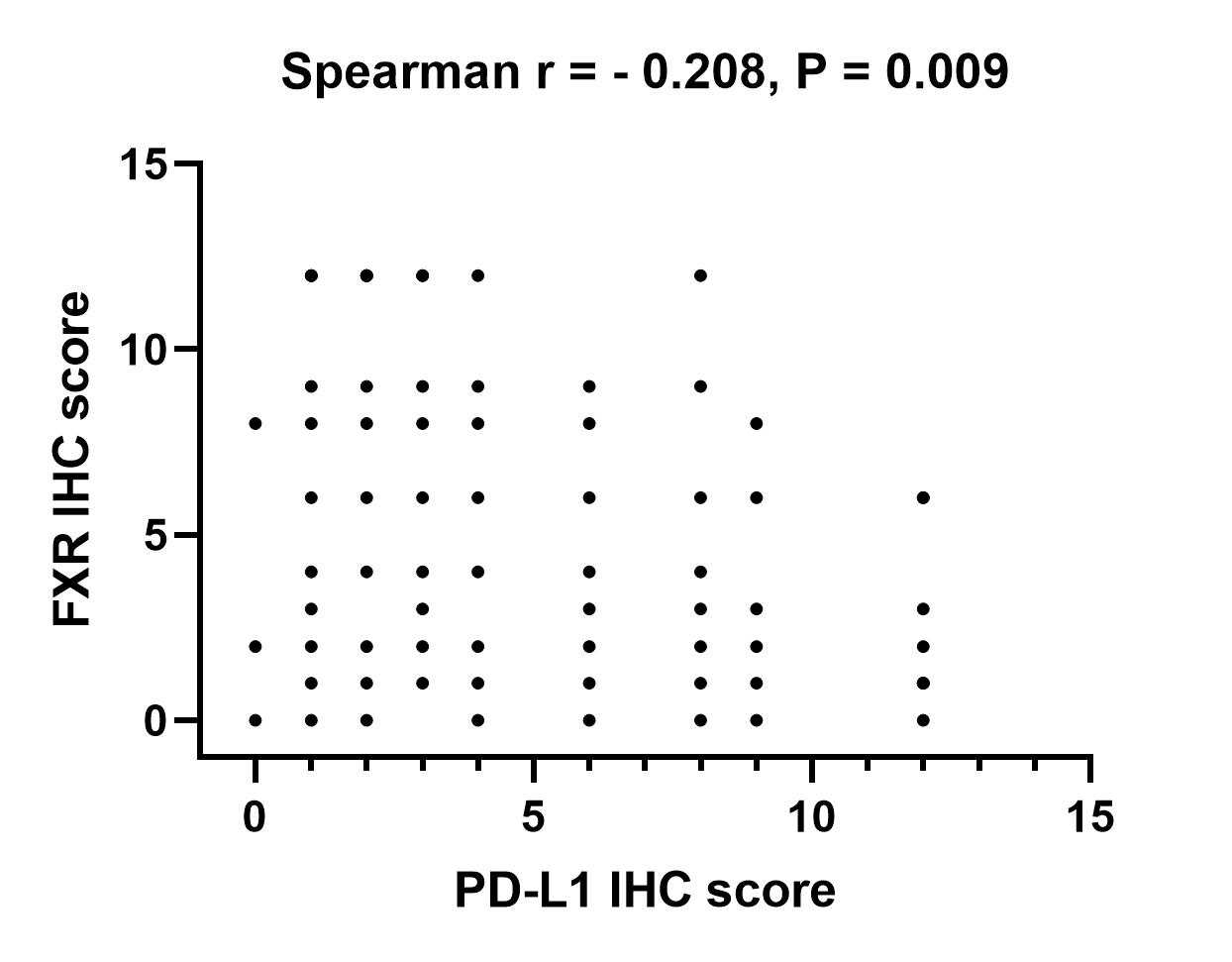


**Legend Supplementary Figure 11**

The spearman correlation analysis between PD-L1 and FXR in the HCC tissues

**Supplementary Table 1**

Primer sequences

| SHP up | GTGCCCAGCATACTCAAGAAG |
| --- | --- |
| SHP down | TGGGGTCTGTCTGGCAGTT |
| BSEP up | GCCGCAGCTCGTCAGATAC |
| BSEP down | GAATTGCAGTCAAACCACCCTAT |
| ABCB4 up | CCTTGTCGCTGCTAAATCCAG |
| ABCB4 down | AGCACCCAATCCTGAGTAGTAA |
| OSTalpha up | TCATTTCCCGTCAAGCCAGG |
| OSTalpha down | GGCGAACAAGCAATCTGCC |
| [CYP3A4 up](https://www.ncbi.nlm.nih.gov/gene/1576) | CACGAGCAGTGTTCTCTCCTT |
| [CYP3A4 down](https://www.ncbi.nlm.nih.gov/gene/1576) | CACAGTATCATAGGTGGGTGGT |
| [FGF19 up](https://www.ncbi.nlm.nih.gov/gene/9965) | CGGAGGAAGACTGTGCTTTCG |
| [FGF19 down](https://www.ncbi.nlm.nih.gov/gene/9965) | CTCGGATCGGTACACATTGTAG |
| FXR up | GACTTTGGACCATGAAGACCAG |
| FXR down | GCCCAGACGGAAGTTTCTTATT |
| GAPDH up | ACAACTTTGGTATCGTGGAAGG |
| GAPDH down | GCCATCACGCCACAGTTTC |

**Supplementary Table 2**

Detection of liver function, kidney function, blood, urine and weight of the mice

|  | nc1 | nc2 | nc3 | nc4 | nc5 | nor1 | nor2 | nor3 | nor4 | nor5 | p |
| --- | --- | --- | --- | --- | --- | --- | --- | --- | --- | --- | --- |
| Weight | 1.1 | 1.9 | 1.0 | 1.3 | 1.4 | 1.3 | 1.2 | 1.4 | 1.2 | 1.2 | n.s. |
| ALP | 103.1 | 106.7 | 111.4 | 95.5 | 107.3 | 92.2 | 110.4 | 103.6 | 95.1 | 97.6 | n.s. |
| AST | 257.2 | 273.2 | 259.2 | 260.2 | 261.2 | 242.2 | 263.2 | 264.2 | 235.2 | 241.2 | n.s. |
| ALT | 103.1 | 118.0 | 107.3 | 112.3 | 117.4 | 120.2 | 110.4 | 103.6 | 95.5 | 95.1 | n.s. |
| TB | 49.5 | 55.5 | 42.9 | 46.5 | 41.4 | 58.2 | 56.3 | 66.7 | 55.1 | 38.2 | n.s. |
| ALB | 32.0 | 33.0 | 34.0 | 35.0 | 36.0 | 36.0 | 38.0 | 39.0 | 40.0 | 31.0 | n.s. |
| WBC | 15.9 | 15.8 | 21.3 | 15.9 | 17.2 | 16.2 | 17.9 | 11.3 | 10.4 | 15.6 | n.s. |
| PLT | 572.0 | 573.0 | 574.0 | 575.0 | 577.0 | 577.0 | 578.0 | 569.0 | 571.0 | 575.0 | n.s. |
| CR | 70.4 | 100.1 | 77.1 | 82.3 | 64.3 | 78.5 | 84.3 | 62.3 | 94.7 | 64.3 | n.s. |
| BUN | 34.0 | 43.7 | 39.5 | 38.8 | 39.2 | 38.7 | 35.1 | 39.0 | 35.8 | 40.1 | n.s. |
| Uri wbc | 0.0 | 0.0 | 0.0 | 70.0 | 0.0 | 70.0 | 0.0 | 0.0 | 0.0 | 0.0 | n.s. |
| Uri TB | 8.6 | 8.3 | 7.9 | 8.6 | 8.6 | 8.6 | 7.0 | 8.2 | 8.3 | 8.6 | n.s. |
